# Y_2_Ti_2_O_5_S_2_ – a promising n-type oxysulphide for thermoelectric applications†

**DOI:** 10.1039/d2ta04160j

**Published:** 2022-07-04

**Authors:** Katarina Brlec, Kieran B. Spooner, Jonathan M. Skelton, David O. Scanlon

**Affiliations:** Department of Chemistry, University College London 20 Gordon Street London UK; Thomas Young Centre, University College London Gower Street London UK; Department of Chemistry, University of Manchester Oxford Road Manchester UK d.scanlon@ucl.ac.uk

## Abstract

Thermoelectric materials offer an unambiguous solution to the ever-increasing global demand for energy by harnessing the Seebeck effect to convert waste heat to electrical energy. Mixed-anion materials are ideal candidate thermoelectric materials due to their thermal stability and potential for “phonon-glass, electron-crystal” behaviour. In this study, we use density-functional theory (DFT) calculations to investigate Y_2_Ti_2_O_5_S_2_, a cation-deficient Ruddlesden-Popper system, as a potential thermoelectric. We use hybrid DFT to calculate the electronic structure and band alignment, which indicate a preference for n-type doping with highly anisotropic in-plane and the out-of-plane charge-carrier mobilities as a result of the anisotropy in the crystal structure. We compute phonon spectra and calculate the lattice thermal conductivity within the single-mode relaxation-time approximation using lifetimes obtained by considering three-phonon interactions. We also calculate the transport properties using the momentum relaxation-time approximation to solve the electronic Boltzmann transport equations. The predicted transport properties and lattice thermal conductivity suggest a maximum in-plane *ZT* of 1.18 at 1000 K with a carrier concentration of 2.37 × 10^20^ cm^−3^. Finally, we discuss further the origins of the low lattice thermal conductivity, in particular exploring the possibility of nanostructuring to lower the phonon mean free path, reduce the thermal conductivity, and further enhance the *ZT*. Given the experimentally-evidenced high thermal stability and the favourable band alignment found in this work, Y_2_Ti_2_O_5_S_2_ has the potential to be a promising high-temperature n-type thermoelectric.

## Abstract 2

A hybrid-DFT study shows Y_2_Ti_2_O_5_S_2_ is a promising earth-abundant thermoelectric with maximum *ZT* of 1.18 at 1000 K.

## Graphical abstract

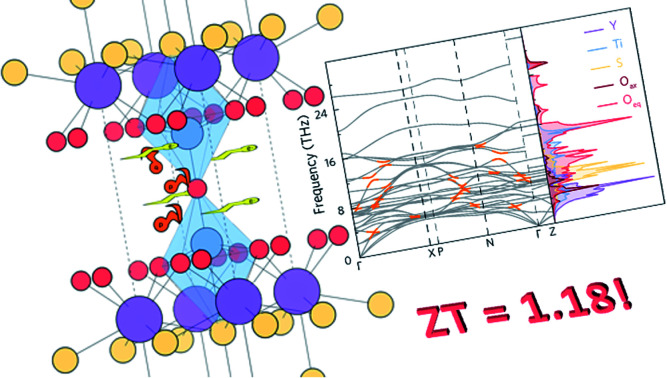

One of the many pathways to mitigating the impact of climate change is to increase the energy efficiency of current systems and processes. The substantial thermal losses from internal combustion engines can be offset by thermoelectric generators, which convert waste heat into useful electrical energy by exploiting the Seebeck effect in a thermoelectric material. The thermoelectric performance of TE materials is evaluated using the figure of merit, *ZT*, which is a dimensionless quantity related to the conversion efficiency:1
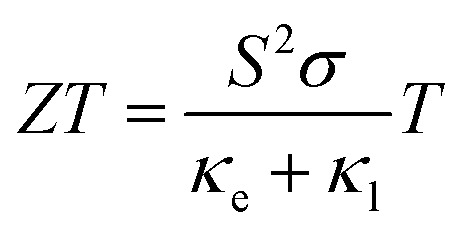
where *S* is the Seebeck coefficient, *σ* is the electrical conductivity, *T* is the absolute temperature, and *κ*_e_ and *κ*_l_ are the electronic and lattice (phonon) components of the thermal conductivity. The best performing materials thus far have achieved *ZT* > 2 in a laboratory setting, including PbTe (2.5 at 973 K) and SnSe (2.6 at 973 K).^1,2^ Ideally, to compete with traditional energy conversion methods, materials with *ZT* > 3 are needed to achieve a 20–30% thermal-to-electrical energy conversion efficiency.

Maximising *ZT* is a complex task due to the interconnected nature of its components. A high Seebeck coefficient is achieved by flat, non-disperse electronic bands with large charge-carrier effective masses.^3^ Conversely, a high electrical conductivity requires high charge-carrier mobility and relatively disperse band edges.^4^ At the same time, high electrical conductivity leads to high electronic thermal conductivity (*κ*_e_ ∝ *σT*). Lattice thermal conductivity is often the limiting factor in candidates that could otherwise challenge the current best-performing thermoelectric materials. Ideally TE materials would show the low thermal conductivity characteristic of an amorphous material (glass) and the high electrical conductivity of a crystal – a “phonon-glass, electron-crystal” material.^5^

In recent years the focus has been on transitioning from traditional thermoelectric materials such as PbTe, Bi_2_Te_3_ and Bi_2_Se_3_, which contain toxic and rare elements, to materials formed from more abundant and environmentally-friendly components. In theory, oxides are a good fit for thermoelectric applications given their high-temperature stability in air, low toxicity and straightforward synthesis paths. Indeed, several p-type oxides with *ZT* exceeding unity have been discovered since 1997, when NaCo_2_O_4_ showed promise as an oxide thermoelectric.^6^ Na_*x*_CoO_2_ and Ca_3_Co_4_O_9_ are currently some of the best ternary p-type thermoelectrics, with *ZT* exceeding 1 at 800 K and 1.1 at 1000 K, respectively.^7,8^ Mixed-anion materials have also proven to be successful, with p-type oxide BiCuOSe exhibiting a high *ZT* of 1.4 at 923 K.^9^ Recently, two oxypnictogens LaZnOP and LaZnOAs were predicted to have theoretical maximum p-type *ZT* scores of 2.10 and 1.92, respectively, at 1000 K.^10^

The perovskite oxides CaMnO_3_, SrTiO_3_ and BaSnO_3_ are among the most well-studied n-type candidates, but their thermoelectric performance currently lags behind their p-type counterparts. For CaMnO_3_, W doping led to a peak *ZT* of 0.15 at 973 K, while co-doped Ca_0.96_Dy_0.2_Yb_0.2_MnO_3_ exhibited a maximum *ZT* of 0.2 at the same temperature.^11,12^ Nb- and La-doped SrTiO_3_ achieved a maximum *ZT* of 0.37 at 973 K, while co-doping with both Nb and La pushed the peak *ZT* to over 0.6 at 1100 K.^13–15^ The transparent conducting oxide BaSnO_3_ has a much higher predicted peak *ZT* of 2.16 at 1700 K, but the predicted *ZT* at a more reasonable and comparable temperature of 1000 K is only 0.62.^16^ Mg_2_(Sb,Bi)_3_ is the only mixed-anion n-type thermoelectric to have been discovered so far, most likely due to the lack of research into mixed anion systems.^17,18^ Nevertheless, such compounds lend themselves well to the phonon-glass, electron-crystal school of thought, as anions of different size would be expected to scatter phonons, leading to naturally low lattice thermal conductivity, which makes such systems a promising avenue of research.

Y_2_Ti_2_O_5_S_2_ was first reported in a study of the structural and optical properties of the Ln_2_Ti_2_O_5_S_2_ series.^19,20^ The material crystallises in the tetragonal space group *I*4/*mmm* (no. 139) in a unique quasi-layered structure consisting of two connected building blocks, *viz.* layers of two-dimensional corner-sharing octahedra and rock-salt layers (Fig. 1). Unlike some layered systems, which are held together by weak dispersion forces, the titanium and yttrium motifs in Y_2_Ti_2_O_5_S_2_ are held together by Ti–S and Y–O bonds. Y_2_Ti_2_O_5_S_2_ has been studied as a potential battery anode but has also proven to be an excellent candidate for hydrogen production by photocatalytic water splitting.^21–26^ The performance of the material in these applications stems from its uniquely complex structure, which renders it stable to Li intercalation and gives rise to relatively disperse electronic bands. The complex structure is also expected to facilitate strong phonon scattering and low lattice thermal conductivity, making Y_2_Ti_2_O_5_S_2_ a promising thermoelectric candidate.

**Fig. 1:**
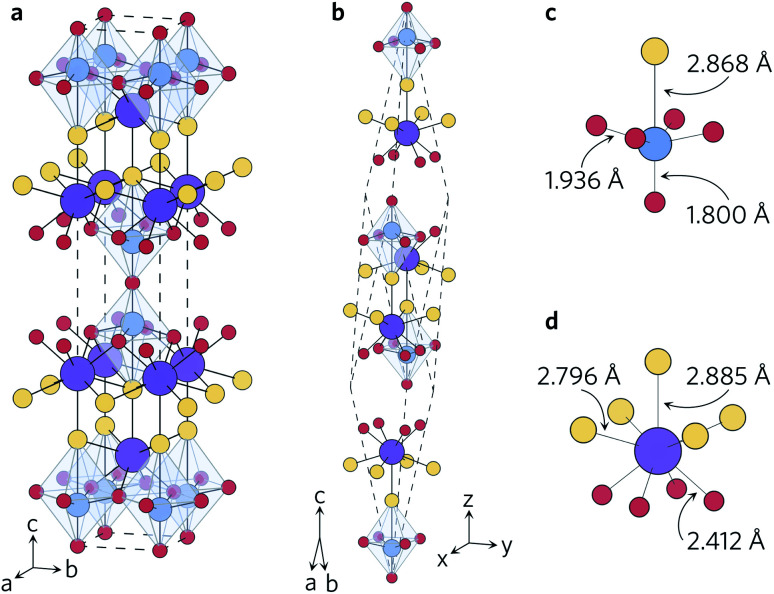
Conventional (a) and primitive (b) unit cells of Y_2_Ti_2_O_5_S_2_ and local bonding environments of the TiO_5_S octahedra (c) and YO_4_S_5_ motifs (d). The atom colours are as follows: Y = purple, Ti = blue, S = yellow, O = red. These images were prepared using VESTA.^40^

## 1 Computational methodology

The calculations in this study were performed using density-functional theory (DFT) as implemented in the Vienna *Ab initio* Simulation Package (VASP) code.^27^ The ion cores were modelled with projector-augmented wave (PAW) pseudopotentials.^28,29^ An energy cutoff of 480 eV and *Γ*-centred k-point meshes with 5 × 5 × 5 and 5 × 5 × 1 subdivisions were chosen respectively to model the valence wavefunctions of the 11-atom primitive and 22-atom conventional unit cell. These parameters were chosen to converge the total energy to within 1 meV atom^−1^. Further information on the convergence testing and input parameter selection is available in the ESI.†

The structures were relaxed using both the Perdew–Burke–Ernzerhof generalised gradient approximation (GGA) functional revised for solids (PBEsol)^30^ and the Heyd–Scuseria–Ernzerhof (HSE06) hybrid functional,^31,32^ with no constraints on the unit-cell shape or size, until the maximum force on any atom did not exceed 0.0005 eV Å^−1^. During these calculations a 30% higher cutoff (620 eV) was employed to account for Pullay stresses.^33^ The electronic band structure and density of states (DOS) were calculated with HSE06 and analysed using the sumo package,^34^ which was also used to evaluate the effective charge-carrier masses using parabolic fitting of the band edges.

To obtain the Seebeck coefficient, electrical conductivity and electronic thermal conductivity, the electronic Boltzmann transport equations were solved using AMSET.^35^ Historically, the constant relaxation-time approximation (CRTA) has been used to solve the transport equations, but it has been shown that the mean absolute percentage error in the charge carrier mobility obtained with a constant relaxation time is unacceptable.^35,36^ AMSET improves on the CRTA by using the momentum relaxation-time approximation (MRTA) to explicitly calculate scattering rates for each electronic state within the Born approximation for a sequence of temperature and charge-carrier concentration couples. In particular, scattering due to the acoustic deformation potential (ADP), ionised impurities (IMP), piezoelectric interactions (PIE) and polar optical phonons (POP) can be evaluated, together with the effect of limiting the phonon mean-free paths by nanostructuring. Details of the parameters used in the AMSET calculations can be found in the SI.

To calculate the second- and third-order force constants (FCs) needed to compute the lattice thermal conductivity, we employed the finite-displacement method as implemented in the Phonopy and Phono3py packages.^37,38^ A 1100-atom 5 × 5 × 2 supercell based on the conventional unit cell was used to compute the second-order FCs, while a smaller 704-atom 4 × 4 × 2 supercell was used to evaluate the third-order FCs. The convergence of the phonon dispersion with respect to the supercell size used to compute the second-order FCs was explicitly checked. In total 19 743 displacements were evaluated to compute the third-order FCs in our chosen 4 × 4 × 2 supercell, using the default displacement distance of 0.03 Å. The PBEsol functional was used for the phonon and thermal-conductivity calculations, as it has been shown to balance computational cost and accuracy to provide a good description of the lattice dynamics.^39^ A non-analytical correction (NAC) to treat the long-range interaction between the charges on the ions and the macroscopic electric field was also included.

The lattice thermal conductivity was calculated by solving the phonon Boltzmann transport equation under the single-mode relaxation-time approximation. The lattice thermal conductivity was converged with respect to the q-point sampling density, resulting in a 25 × 25 × 25 mesh being selected (ESI Fig. S8†).

## 2 Results

### 2.1 Crystal structure

Y_2_Ti_2_O_5_S_2_ crystallises in the body-centred tetragonal space group *I*4/*mmm* (no. 139) with the conventional and needle-like primitive unit cells shown in Fig. 1. In the latter cell the lattice lengths are the same, *i.e. a* = *b* = *c*, and *α* = *β* > 120° while *γ* is acute. The following transformation matrix was used to convert the conventional unit cell to the primitive unit cell:2
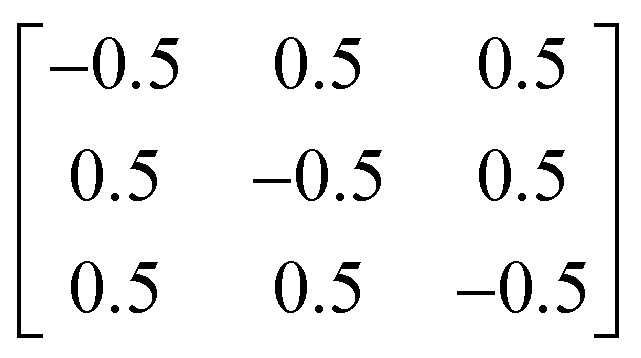


The lattice parameters of the conventional unit cell, optimised using both PBEsol and HSE06, are listed and compared to experimental measurements and other calculations in Table 1.

**Table 1:** Lattice parameters of the conventional unit cell of Y_2_Ti_2_O_5_S_2_ obtained from the PBEsol and HSE06 calculations performed in this work, compared with experimental results from Hyett *et al.* and calculations by McColl and Corá and the Materials project (MP).^22,41,42^ (Materials project data accessed April 2020.)

	*a*/Å	*c*/Å
PBEsol	3.755	22.683
HSE06	3.756	22.871
Hyett *et al.* (exp.)	3.7696	22.8056
McColl and Corá (PBEsol)	3.7593	22.7233
Materials project (PBE)	3.8001	23.071

The calculated lattice parameters closely agree with measurements, with the volume of the relaxed unit cell decreasing by only 1.3% (PBEsol) and 0.4% (HSE06) relative to the experimental structure. The structural relaxations with both functionals generally shortened the Y–O and Y–S bonds in the YO_4_S_5_ motif (Fig. 1d), with the exception that the axial Y–S bond was elongated by 0.02 Å in the HSE06 optimisation. The equatorial Ti–O bonds in the TiO_5_S octahedra (Fig. 1c) were shrunk by 0.006 Å, while the axial relaxation differed between the two functionals such that in the PBEsol-relaxed structure the axial Ti–O and Ti–S bond lengths increased and decreased, respectively, whereas the opposite was observed in the HSE06-relaxed structure. The greater volume contraction in the PBEsol-relaxed structure can be predominantly attributed to the axial Ti–S and Y–S bonds shortening by 0.021 Å and 0.048 Å, respectively, whereas the lengths of these two bonds were increased by 0.022 Å and 0.017 Å in the HSE06 structure.

### 2.2 Electronic structure

**Fig. 2:**
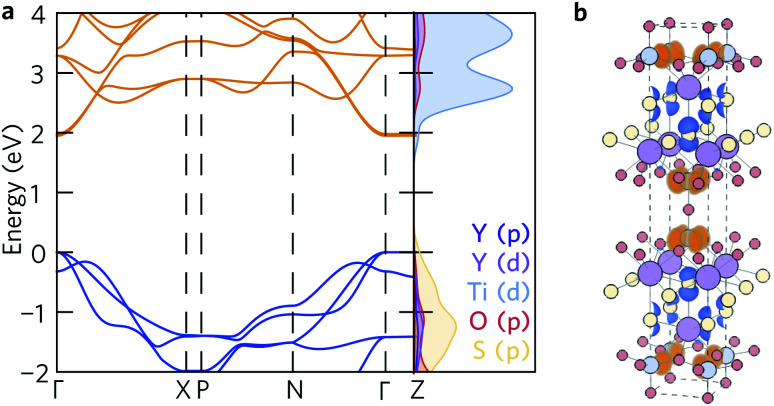
(a) Calculated electronic band structure of Y_2_Ti_2_S_2_O_5_ along the Bradley–Cracknell-derived k-point path,^43^ with the corresponding atom-projected (partial) density of states. This figure was prepared using sumo.^34^ (b) Partial charge density of the valence band maximum (blue) and conduction band minimum (orange) projected onto the structure. The atom colours are as follows: Y = purple, Ti = blue, S = yellow, O = red. This image was prepared using VESTA.^40^

The density of states (Fig. 2a) agrees well with previous reports – the conduction band is dominated by S and O states, and the valence band is primarily Ti in character. This is further corroborated by the spatial separation of the two conduction channels observed by projecting the valence-band maximum (VBM, in blue) and conduction-band minimum (CBM, in orange) onto the crystal structure (Fig. 2b). The VBM is composed only of S p_*x*_ orbitals, confirming that the overlapping O and S peaks in the density of states do not indicate S 3p and O 2p hybridisation at the band edge. Likewise, the CBM is composed of Ti d_*xy*_ orbitals, with no significant hybridisation to O 2p or S 3p states.

The fundamental band gap of 1.95 eV calculated using hybrid DFT occurs at *Γ* and is in line with the experimental band gaps of 1.9 eV to 2.0 eV.^20,25^ The band gaps of 2.16 eV and 2.19 eV obtained by McColl and Corà, using HSE06 with and without van der Waals dispersion corrections, respectively, are slightly greater.^22^ It is worth nothing, however, that these calculations did not use a plane-wave basis set and computed the band structure based on the k-point path for a simple rather than body-centred tetragonal system.

The electronic band structure (Fig. 2a) exhibits similar curvature near both the valence- and conduction-band edges. The charge-carrier effective masses at the band edges are tabulated in Table 2. The band structure is highly anisotropic with respect to the in-plane (*xy*) and out-of-plane (*z*) directions. The bands along the Γ–Z and N–P directions, which correspond to the *z* direction in real space, are flat and heavy, with effective electron and hole masses of 84 *m*_e_ along the Γ–Z direction calculated from parabolic fits of the band edges. Conversely, the bands associated with the in-plane directions are more disperse and several orders of magnitude lighter at the band edges, resulting in effective electron masses of 0.279 *m*_e_ (Γ–X) and 0.312 *m*_e_ (Γ–N) and hole masses of 0.538 *m*_e_ and 0.554 *m*_e_ along the same directions.

**Table 2:** Charge-carrier effective masses calculated from parabolic fits of the band edges performed using sumo^34^

Charge carrier	Direction	Effective mass (*m*_e_)
e^−^	Γ–X (*xy*)	0.538
e^−^	Γ–N (*xy*)	0.554
e^−^	Γ–Z (*z*)	84
h^+^	Γ–X (*xy*)	0.279
h^+^	Γ–N (*xy*)	0.312
h^+^	Γ–Z (*z*)	84

An extended band structure was calculated using the k-point path from the SeeK-path formalism^44^ to include the Γ–S_0_ path, which corresponds to the *x*-direction in real space (see ESI Fig. S6†). The band structure along this path is similar to that along Γ–N, with a minor difference in the −2 eV to −4 eV region of the valence band. The calculated effective hole and electron masses of 0.301 *m*_e_ and 0.537 *m*_e_ are in line with the other *xy*-plane effective masses.

**Fig. 3:**
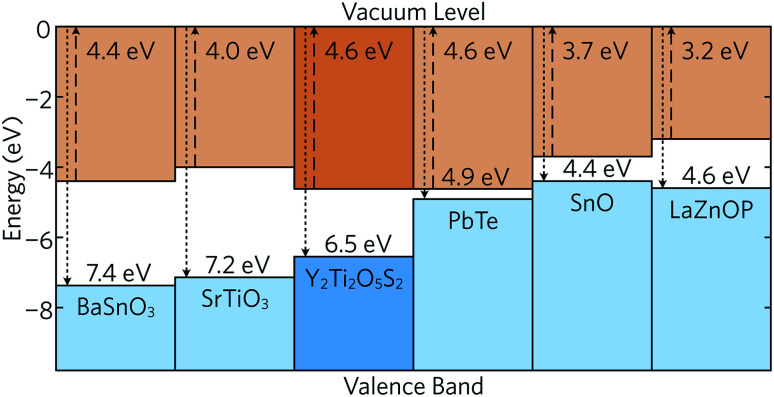
Band alignment of Y_2_Ti_2_O_5_S_2_ calculated in this study and compared to the band alignments of n-type BaSnO_3_^46^ and SrTiO_3_,^46^ bipolar PbTe^47^ and SnO,^48^ and p-type LaZnOP.^10^

The band alignment of bulk Y_2_Ti_2_O_5_S_2_ was also calculated using the core and vacuum energies from the (001) bulk-like surface slab using the method developed by Wei and Zunger (see ESI for more details†)^45^ and is shown in Fig. 3 together with the band alignments of several other high-performance thermoelectrics. The electron affinities (EA) and ionisation potentials (IP) can give an indication of the probability for p- or n-type dopability. Y_2_Ti_2_O_5_S_2_ exhibits a relatively large IP, comparable to those of n-type BaSnO_3_ and SrTiO_3_, which indicates that the formation of p-type defects is unlikely. The IP of Y_2_Ti_2_O_5_S_2_ is also much greater than that of bipolar PbTe and SnO and p-type LaZnOP. The calculated EA is high and again similar to those of BaSnO_3_ and SrTiO_3_, which provides further evidence that Y_2_Ti_2_O_5_S_2_ is likely to prefer n-type behaviour.

Due to the significant anisotropy in the electronic structure, it is expected that charge carrier mobility in the *z* direction is significantly impeded. On the other hand, the low effective masses of holes and electrons are predicted to allow for sufficiently good in-plane mobility and conductivity, assuming the material is both n- and p-type dopable. However, the band alignment suggests Y_2_Ti_2_O_5_S_2_ favours n-type doping over p-type as per doping limit rules.^49^ Consequently, here we have focused our investigation on the n-type performance of Y_2_Ti_2_O_5_S_2_.

### 2.3 Lattice dynamics

**Fig. 4:**
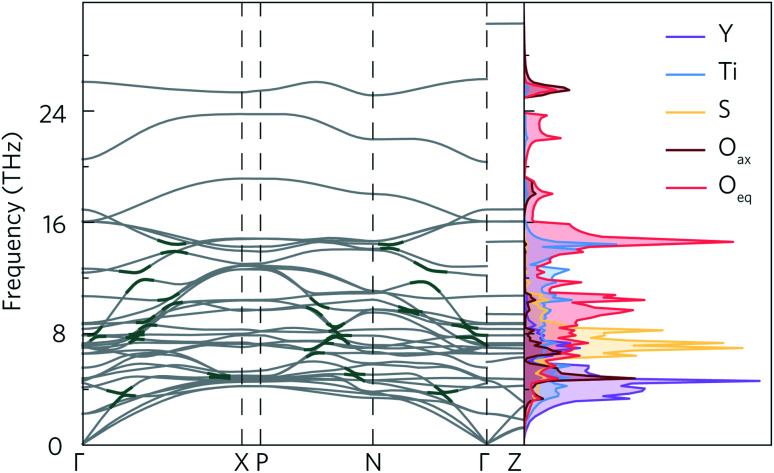
Calculated phonon dispersion for a 5 × 5 × 2 supercell with non-analytical corrections applied (NAC) and atom-projected density of states of Y_2_Ti_2_O_5_S_2_. Some of the avoided crossings marked in dark green. Phonon dispersion was plotted using ThermoPlotter,^51^ and the high-symmetry paths were constructed using the Bradley–Cracknell formalism.^43^

The calculated phonon dispersion of Y_2_Ti_2_O_5_S_2_ (Fig. 4) contains no imaginary modes, indicating the system is dynamically stable. The same was true for all of the supercell sizes tested (ESI Fig. S7†). The dispersion covers a large range of frequencies, with the highest frequency modes reaching 30 THz. The atom-projected (partial) phonon density of states (PDOS) shows that the greatest contribution to modes at frequencies below 8 THz comes from motion of the yttrium atoms, which is expected due to the inverse relationship between frequency and atomic mass. Between 6 THz to 10 THz, sulphur motion dominates, while titanium contributions are observed across the whole frequency spectrum. Somewhat surprisingly, oxygen motion also contributes significantly to phonon modes across the spectrum, even at low frequencies. Moreover, both of the symmetry-inequivalent oxygen atoms contribute to the low-frequency modes. A similar phenomenon, where Ti and O motion contribute to modes over a large range of frequencies, including lower frequencies, has been observed in anatase TiO_2_, SrTiO_3_ and BaTiO_3_.^50^ All three structures have in common that they contain TiO_6_ octahedral structural motifs, so it is likely that the low-frequency oxygen modes in Y_2_Ti_2_O_5_S_2_ originate from the TiO_5_S octahedra.

As for the electronic band structure, the phonon dispersion shows notable anisotropy between the in-plane and out-of-plane directions. The inclusion of NAC has a particularly striking effect along the out-of-plane direction between 26 THz to 30 THz where the continuity of the band is broken leading to a marked flattening of the band. With the exception of the three acoustic modes along Γ–Z, the bands along the X–P and Γ–Z directions, which correspond to the *z* direction in real space, are almost completely flat (*i.e.* dispersion-less). As the group velocity is calculated from the band curvature, this flattening is expected to lower the velocity, which would favour low lattice thermal conductivity. The effect of NAC is less pronounced in the *xy* plane, but flattening of the modes be seen between 8 THz to 12 THz and 16 THz to 24 THz along Γ–X and Γ–N, where the dispersion curvature levels out close to the high-symmetry wavevectors.

Avoided crossings in the phonon dispersion tend to “depress” the phonon frequencies and lower the band curvature and group velocities, thereby also lowering the lattice thermal conductivity. Such features typically indicate the presence of “rattling” species, such as heavy filler atoms in the cavities in, for example, skutterudites and clathrates.^52^ With a quasi-layered structure and fairly light constituent atoms, rattling behaviour would not be expected in Y_2_Ti_2_O_5_S_2_. However, several avoided crossings can be seen along the Γ–X, P–N and Γ–N paths in the phonon dispersion, both between the optic and acoustic modes from 0 THz to 4 THz and also between different optic modes in the 6 THz to 16 THz range (Fig. 4).

**Fig. 5:**
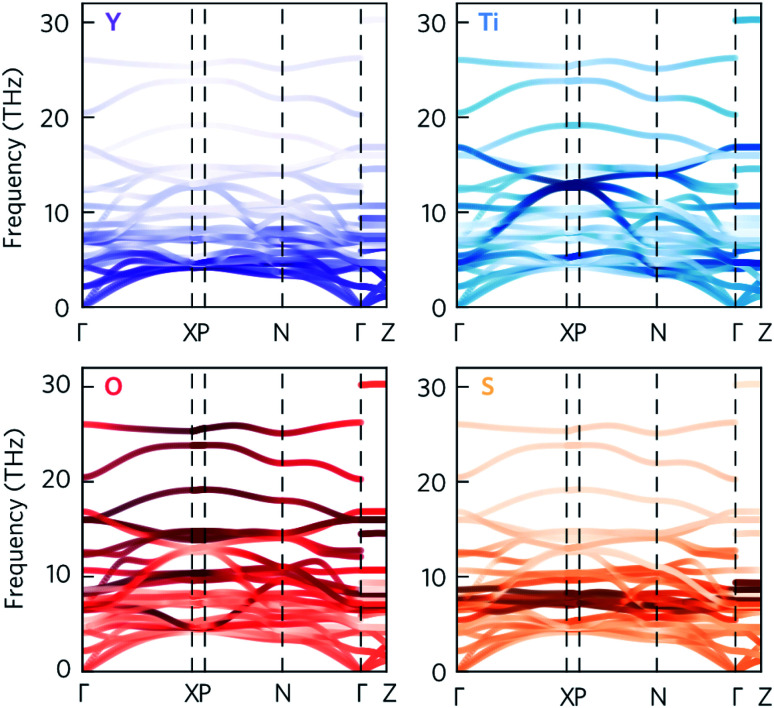
Phonon dispersion of Y_2_Ti_2_O_5_S_2_, calculated using a 5 × 5 × 2 supercell, showing the projection of the eigen-displacements onto the Y, Ti, O, and S atoms. The darker the colour, the greater the contribution from those atoms. The high-symmetry paths were created using the Bradley–Cracknell formalism.^43^

Given that the Y_2_Ti_2_O_5_S_2_ structure would not necessarily be expected to show avoided crossings, it is worth exploring where these features originate from. The atom-projected phonon density of states would normally be used to elucidate which clusters of bands originate from which species. However, as the majority of the avoided crossings are between 6 THz to 14 THz, where the PDOS shows overlap between multiple atom types (Fig. 4a), it is not possible to tell the different contributions apart. We therefore instead projected the elemental eigen-displacements, which can be interpreted as the extent to which atoms are displaced along the mode, onto the phonon dispersion (Fig. 5). This analysis shows which atomic displacements contribute most to the phonon dispersion at each band and wavevector.

The change in mode character along the avoided crossing in the disperse Ti mode from 8 THz to 12 THz between Γ–X and P–N is particularly striking. The mode crosses a number of relatively flat modes associated primarily with S and O movement, resulting in an abrupt change in character in particular around 8 THz and 10 THz where the S and O modes dominate the PDOS. The two highest-frequency avoided crossings highlighted in Fig. 4 at just above 14 THz similarly result from Ti rattling against the equatorial O, while the two crossings involving the Y-dominated acoustic modes are to avoid an interaction with the axial O (see ESI Fig. S9†). The avoided crossings with the largest frequency gaps are difficult to assign to the movements of particular atoms, so we tentatively ascribe them to symmetry-forbidden crossings.

**Fig. 6:**
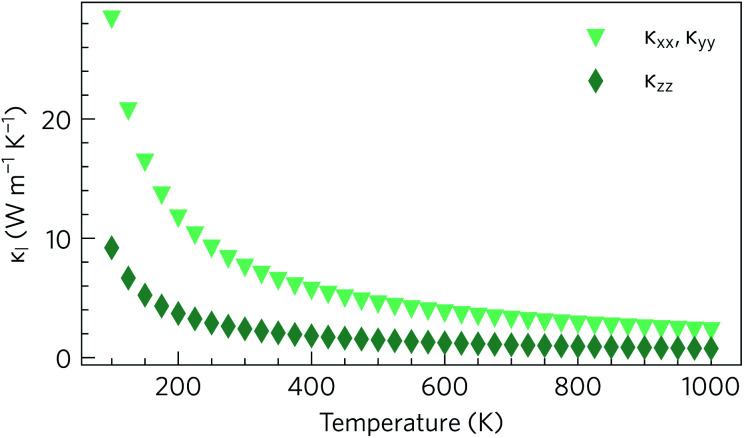
Calculated lattice thermal conductivity *κ*_l_ of Y_2_Ti_2_O_5_S_2_ as a function of temperature, shown separately in the *xy* plane (*κ*_*xx*_, *κ*_*yy*_) and along the *z* direction (*κ*_*zz*_).

As expected based on the crystal structure and phonon dispersion, the lattice thermal conductivity is anisotropic. However, in this case the anisotropy favours a lower thermal conductivity along the *z* direction, where the variation in the size and flavour of structural motifs is larger, and a higher thermal conductivity through the quasi-layers in the *xy* plane, where the electronic structure predicts the best transport properties. The calculations predict an in-plane thermal conductivity of 2.27 W m^−1^ K^−1^ at 1000 K, compared to a much smaller 0.76 W m^−1^ K^−1^ in the *z* direction (Fig. 6). At all temperatures, the in-plane lattice thermal conductivity is at least twice the out-of-plane conductivity, *e.g.* at 300 K the thermal conductivities are 7.59 W m^−1^ K^−1^ and 2.41 W m^−1^ K^−1^ respectively. Nonetheless, it can be seen that the 300 K lattice thermal conductivity of Y_2_Ti_2_S_5_O_2_, particularly the out-of-plane component, is competitive with or lower than that of other high-performance n-type thermoelectrics such as SrTiO_3_ (11 W m^−1^ K^−1^), PbTe (4 W m^−1^ K^−1^) and BaBi_2_O_6_ (3.6 W m^−1^ K^−1^).^36,53,54^

### 2.4 Transport properties

**Fig. 7:**
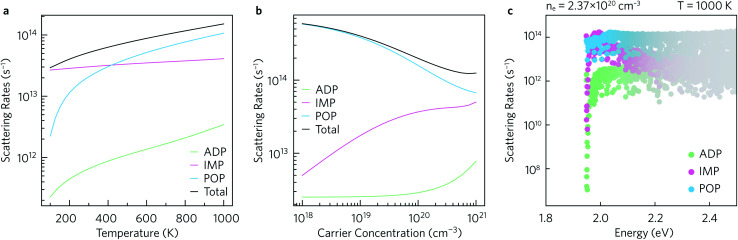
Average scattering rates (a) as a function of temperature for a fixed *n* = 2.37 × 10^20^ cm^−3^, and (b) as a function of charge carrier concentration for a fixed *T* = 1000 K. The scattering rates as a function of energy at *n* = 2.37 × 10^20^ cm^−3^ and *T* = 1000 K, weighted by the derivative of the Fermi–Dirac distribution, are shown in (c). Acoustic deformation potential (ADP) scattering rates are shown in green, ionised impurity scattering rates (IMP) in pink, and polar optical phonon (POP) scattering rates in blue.

The average scattering rates as a function of temperature for a fixed carrier concentration of 2.37 × 10^20^ cm^−3^, and as a function of carrier concentration for a fixed *T* = 1000 K are shown in Fig. 7a and b, respectively. Polar optical phonon (POP) scattering dominates above 425 K, whereas ionised impurity (IMP) scattering, which is comparatively constant across the temperature range, contributes the most to the total scattering below 425 K. As a result of this temperature dependence the POP scattering is dominant at all the charge carrier concentrations shown in Fig. 7b. The IMP scattering rate increases up to carrier concentrations of ∼10^20^ cm^−3^, above which which it largely begins to plateau. Acoustic deformation potential (ADP) scattering contributes the least to the overall scattering rate and is consistently at least one order of magnitude smaller than the total scattering rate, which can be explained by the relatively low deformation potentials of 1.6 eV in the *xy* plane and 0.7 eV along the *z* directions.

The scattering rates can also be examined as a function of energy. Fig. 7c shows the normalised scattering rates at *n* = 2.37 × 10^20^ cm^−3^ and *T* = 1000 K (similar plots at 300 K and 600 K are shown in ESI Fig. S10†). The scattering rates are weighted by the derivative of the Fermi–Dirac distribution so that the darker the colour, the greater the effect of the scattering on the calculated transport properties. In line with previous observations, at the conduction band edge ADP scattering contributes the least to the overall scattering rate, with POP and IMP scattering being the two main scattering mechanisms. Right at the band edge, the POP scattering rates are several orders of magnitude higher than the IMP rates, but the contributions from both are similar between 2.0 eV to 2.1 eV and thus both contribute to the scattering under optimal doping conditions.

**Fig. 8:**
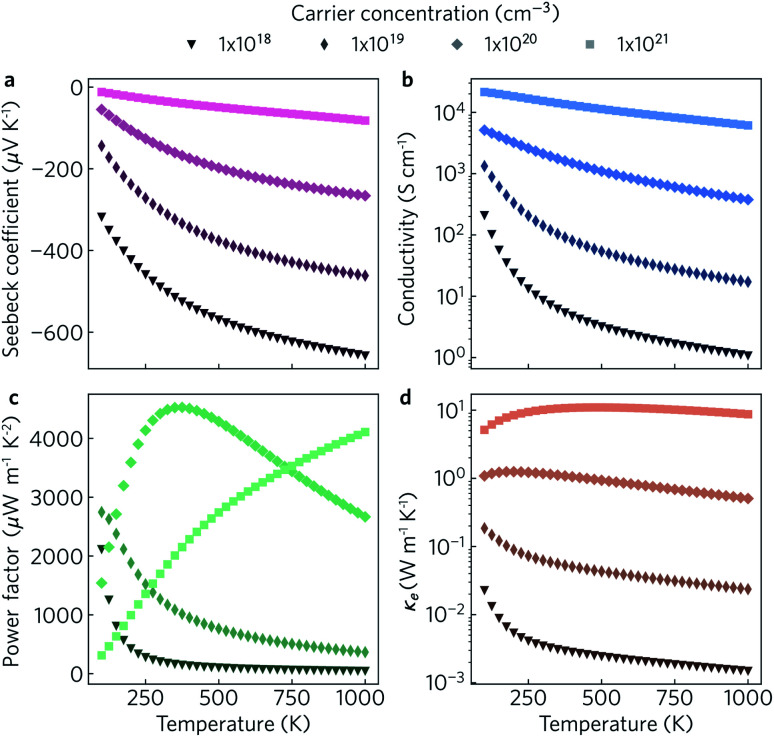
Predicted in-plane Seebeck coefficient *S* (a), electrical conductivity *σ* (b), power factor *S*^2^*σ* (c) and electrical thermal conductivity *κ*_e_ (d) as a function of temperature for charge carrier concentrations of *n* = 1 × 10^18^ cm^−3^, 1 × 10^19^ cm^−3^, 1 × 10^20^ cm^−3^ and 1 × 10^21^ cm^−3^.

**Fig. 9:**
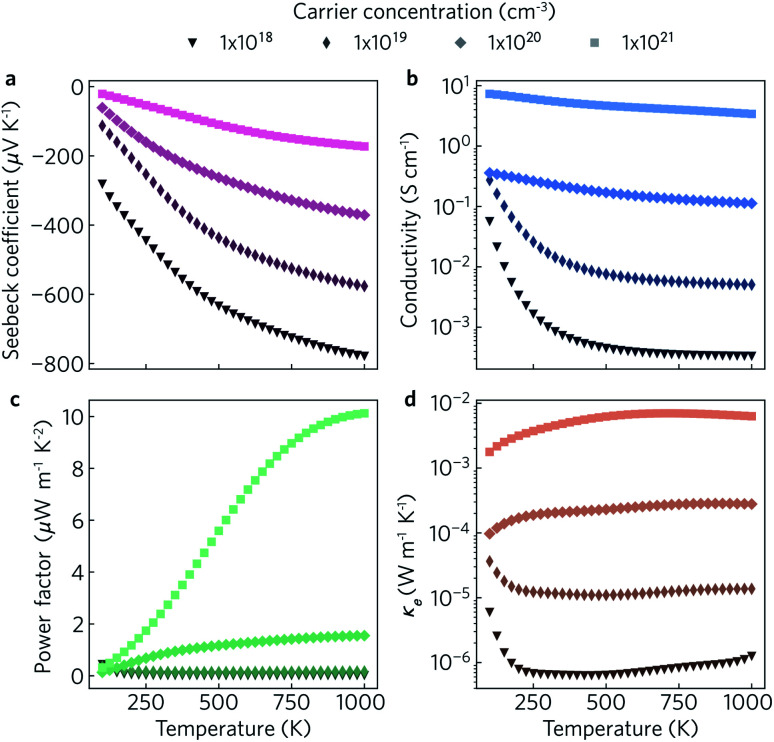
Predicted out-of-plane Seebeck coefficient *S* (a), electrical conductivity *σ* (b), power factor *S*^2^*σ* (c) and electrical thermal conductivity *κ*_e_ (d) as a function of temperature for charge carrier concentrations of *n* = 1 × 10^18^ cm^−3^, 1 × 10^19^ cm^−3^, 1 × 10^20^ cm^−3^ and 1 × 10^21^ cm^−3^.

The electronic transport properties were calculated for n-type doping with carrier concentrations in the range of 10^18^ cm^−3^ to 10^21^ cm^−3^ and temperatures between 100 K and 1000 K. The variation of the Seebeck coefficient, electrical conductivity, power factor and electronic thermal conductivity with temperature for carrier concentrations of 1 × 10^18^ cm^−3^, 1 × 10^19^ cm^−3^, 1 × 10^20^ cm^−3^, and 1 × 10^21^ cm^−3^ in the *xy* plane and along the *z* direction can be seen in Fig. 8 and 9, respectively. We limit the present discussion to n-type doping, as the band alignment suggests n-type behaviour, but calculations for both n- and p-type transport (including scattering rates) can be found in the Zenodo repository.

Of the four electrical properties, the Seebeck coefficient exhibits the least anisotropy. With n-type doping the Seebeck coefficient is negative and shows a gradual decrease with temperature. For both the in-plane and out-of-plane directions *S* follows the expected trends, with the largest doping concentrations leading to least negative values (*i.e.* smaller absolute values). The Seebeck coefficients in the *xy* plane span ranges of −657 μV K^−1^ to −422 μV K^−1^ at *n* = 1 × 10^18^ cm^−3^ and −82 μV K^−1^ to −23 μV K^−1^ at *n* = 1 × 10^21^ cm^−3^. In the *z* direction, more negative Seebeck coefficients are obtained ranging from −778 μV K^−1^ to −396 μV K^−1^ at *n* = 1 × 10^18^ cm^−3^ and −173 μV K^−1^ to −43 μV K^−1^ at *n* = 1 × 10^21^ cm^−3^. This can be ascribed to the shallow curvature and degeneracy of the bands along the out-of-plane direction, as the Seebeck coefficient *S* is proportional to the number of band extrema (*N*_v_) and effective electron mass (m*) and inversely related to the carrier concentration (*n*) *via*
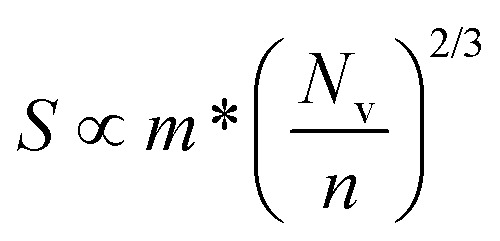
.

The anisotropy between the *xy* plane and *z* direction is much more pronounced in the conductivity, power factor and electronic thermal conductivity (Fig. 8 and 9b–d). This results from the highly-anisotropic effective charge carrier masses discussed in Section 2.2. *σ* is directly proportional to the mobility *μ* through *σ* = *n*e*μ*, where e is electron charge. *μ* is directly related to the scattering relaxation time *τ* and inversely proportional to the m* through 
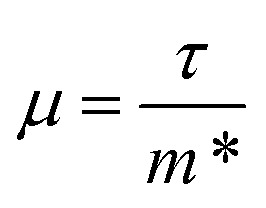
. The conductivities span a range of 1.07 × 10^2^ S m^−1^ to 1.8 × 10^6^ S m^−1^ in the *xy* plane and 0.03 S m^−1^ to 645 S m^−1^ along the *z* direction. Due to the combined effects of stronger scattering and the large m*, the out-of-plane conductivity is three orders of magnitude smaller than in-plane, which directly affects the power factor and electronic thermal conductivity, both of which are directly proportional to *σ*.

The thermoelectric figure of merit is proportional to the power factor *S*^2^*σ* (PF). In the out-of-plane direction, below *n* = 1 × 10^20^ cm^−1^ the PF does not exceed 1 μW m^−1^ K^−2^, and it barely reaches 10 μW m^−1^ K^−2^ at *n* = 1 × 10^21^ cm^−1^. On the other hand, the in-plane power factor spans a range of 46 μW m^−1^ K^−2^ to 6030 μW m^−1^ K^−2^, peaking at *n* = 3.2 × 10^20^ cm^−1^ and *T* = 600 K. The highest low-temperature (<400 K) PFs of ∼3600 μW m^−1^ K^−2^ to 5500 μW m^−1^ K^−2^ are achieved at a carrier concentration of ∼2 × 10^20^ cm^−3^. At intermediate temperatures (400 K to 700 K), the maximum PFs of ∼5600 μW m^−1^ K^−2^ to 6000 μW m^−1^ K^−2^ are obtained for *n* = 3.2 × 10^20^ cm^−3^. At high temperatures (>700 K), the maximum PFs of ∼5400 μW m^−1^ K^−2^ to 5900 μW m^−1^ K^−2^ occur at *n* = 4.2 × 10^20^ cm^−3^.

**Fig. 10:**
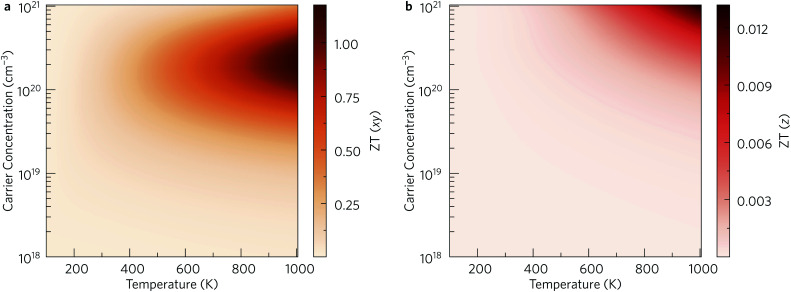
Predicted n-type thermoelectric figure of merit *ZT* in the *xy* plane (a) and along the out-of-plane *z* direction (b) for carrier concentrations between 10^18^ cm^−3^ to 10^21^ cm^−3^ and temperatures between 100 K to 1000 K. This analysis was performed using ThermoPlotter.^51^

Finally, the thermoelectric figure of merit (*ZT*) was calculated using the ThermoPlotter software by combining the calculated electronic transport properties and lattice thermal conductivity.^51^ The predicted in-plane and out-of-plane n-type *ZT* for charge carrier concentrations in the range of 10^18^ cm^−3^ to 10^21^ cm^−3^ and temperatures between 100 K to 1000 K are shown in Fig. 10. This temperature range was chosen based on previous experimental work in which Y_2_Ti_2_O_5_S_2_ was shown to be thermally stable up to 700 °C in air and under an N_2_ atmosphere.^25^

The differences in the in-plane and out-of-plane *ZT* values is striking, with the maximum *ZT* along the *z* direction barely reaching 0.01 at the highest temperature/carrier concentration pairing. On the other hand, the maximum in-plane *ZT* is predicted to be 1.18 at *T* = 1000 K and *n* = 2.37 × 10^20^ cm^−3^. While this is comparable to current state-of-the-art n-type thermoelectrics such as PbTe, it is worth noting that the required charge-carrier concentration is on the higher end of what is achievable for a semiconductor. Table 3 lists the maximum *ZT* and associated carrier concentrations, power factors, and lattice thermal conductivities at 300 K, 600 K and 1000 K. Although the power factor is greater at 600 K than at 1000 K, the comparatively higher lattice thermal conductivity at this temperature leads to a smaller overall *ZT*.

**Table 3:** Predicted maximum in-plane *ZT* at low (300 K), intermediate (600 K) and high (1000 K) temperatures together with the associated charge carrier concentrations (*n*), power factors (PF) and lattice thermal conductivities (*κ*_l_)

*T* (K)	*ZT* in *xy*	*n* (cm^−3^)	PF (μW m^−1^ K^−2^)	*κ*_l_ (W m^−1^ K^−2^)	*κ*_e_ (W m^−1^ K^−2^)
300	0.15	1.33 × 10^20^	4673	7.59	1.61
600	0.58	1.78 × 10^20^	5287	3.77	1.74
1000	1.18	2.37 × 10^20^	4556	2.27	1.60

## 3 Discussion

In general, the *ZT* can be improved either by increasing the power factor or by decreasing the thermal conductivity. Increasing the power factor typically entails increasing the conductivity, which in turn increases the electronic thermal conductivity; since *ZT* is inversely proportional to the sum of the electronic and lattice thermal conductivities, this can effectively negate any improvements to the power factor. In this section, we therefore focus instead on the lattice thermal conductivity. We first establish the microscopic origin of the low lattice thermal conductivity in Y_2_Ti_2_O_5_S_2_ by examining the contributions of individual phonon modes, and we then explore the effect of nanostructuring which, together with alloying, is a common approach to reducing the thermal conductivity and optimising *ZT*.

### 3.1 Microscopic analysis of the lattice thermal conductivity

Within the single mode relaxation time approximation, the lattice thermal conductivity *κ*_l_ is calculated from a sum of contributions from individual phonon modes *λ* as:3
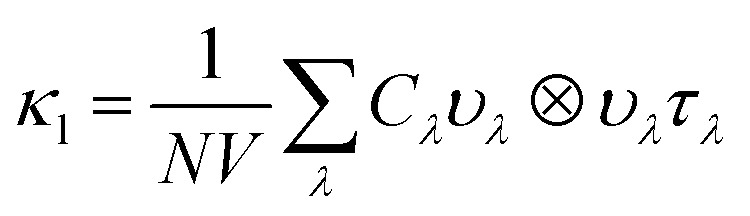
where *N* is the number of wavevectors in the summation (equivalent to the number of unit cells in the crystal), *V* is the unit-cell volume, *C*_*λ*_ are the modal heat capacities, *υ*_*λ*_ are the modal group velocities and *τ*_*λ*_ are the phonon lifetimes. The contributions of individual modes to *κ*_l_ can be examined from scatter plots of the three modal quantities in eqn (3) (*i.e. C*_λ_, *υ*_λ_, *τ*_λ_) against the phonon frequency, coloured according to the modal contributions to *κ*_l_ (*κ*_λ_; the summand in eqn (3)). The effect of the *C*_λ_ on the *κ*_l_ is not considered here, as this quantity is a shallow function of frequency and the variation between phonon modes is therefore minimal.

**Fig. 11:**
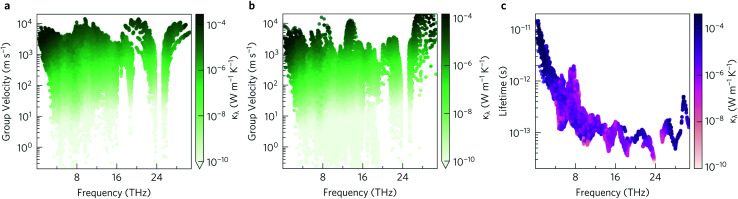
Modal group velocities *υ*_λ_ in the *xy* plane (a) and along the *z* direction (b) and phonon lifetimes (c) as a function of frequency. The lifetimes are calculated at *T* = 1000 K. The modal contributions to the lattice thermal conductivity *κ*_λ_ at 1000 K are overlaid as a colour scale so that the darker the colour the greater the contribution of the mode to the lattice thermal conductivity. Plotted with ThermoPlotter.^51^

The group velocities were previously discussed in the context of the phonon dispersion in Section 2.3. Fig. 11a and b show the *υ*_λ_ in the *xy* plane and along the *z* direction, respectively. In both directions the group velocities fall into the range of 1 m s^−1^ to 1 × 10^4^ m s^−1^. However far more of the modes have in-plane *υ*_λ_ in the range of 10^3^ m s^−1^ to 10^4^ m s^−1^. The fastest modes have in-plane *υ*_λ_ in excess of 3000 m s^−1^ and are present across the entire frequency range, while in *z* direction the fastest modes are limited to the 1 THz to 4 THz, 8 THz to 10 THz, 13 THz to 16 THz and 26 THz to 30 Hz frequency bands. The higher proportion of modes with lower group velocities in the *z* direction would account for the lower out-of-plane *κ*_l_.

The phonon lifetimes are inversely proportional to the phonon linewidths *Γ*_*λ*_*via*:4
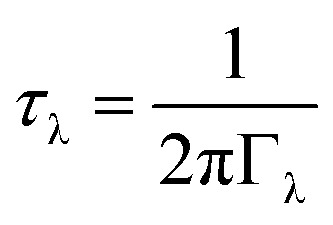
where the *Γ*_λ_ are derived by considering energy- and momentum-conserving three-phonon scattering processes and obtained from the imaginary part of the phonon self-energy. Unlike the group velocities, the lifetimes are scalar quantities. The mode lifetimes are plotted against frequency, with the contributions to *κ*_λ_ overlaid as a colour scale, in Fig. 11c. The lifetimes span a range of 0.1 ps to 10 ps, with over 30% of modes possessing lifetimes longer than 1 ps, even at 1000 K. In general, long lifetimes are indicative of weak phonon scattering and thus weak bond anharmonicity, which is expected in Y_2_Ti_2_O_5_S_2_ due to the similar ion sizes. This is further evident from the average mode Grüneisen parameter of 0.65, another measure of anharmonicity, which is low compared to PbTe (1.96), SrTiO_3_ (1.46) and BiCuOSe (1.5).^53,55,56^ Bond anharmonicity in Y_2_Ti_2_O_5_S_2_ is thus unlikely to be a major contributor to its low lattice thermal conductivity.

**Fig. 12:**
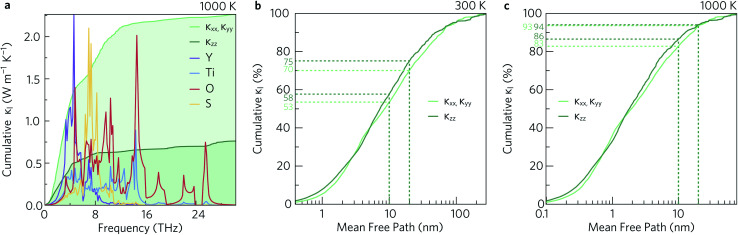
Cumulative lattice thermal conductivity in the *xy* plane (*κ*_*xx*_, *κ*_*yy*_) and along the *z* direction (*κ*_*zz*_) as a function of frequency at *T* = 1000 K compared to the atom-projected density of states (a), and as a function of the phonon mean free paths at *T* = 300 K (b) and *T* = 1000 K (c). The percentages of phonons with mean free paths shorter than 10 nm and 20 nm are marked with dotted lines. These analyses were performed using ThermoPlotter.^51^

To investigate which types of vibration contribute most to the *κ*_l_, the cumulative in-plane and out-of-plane *κ*_l_ as a function of frequency was calculated at 1000 K (Fig. 12a) and compared to the atom-projected density of states. It is clear from this analysis that the majority of the heat transport occurs through the acoustic and low-frequency optic modes, as half of the contributions to the *κ*_l_ in both directions are from modes with frequencies up to 4 THz (This conclusion was found to be independent of temperature). Between 5 THz to 10 THz the cumulative contribution plateaus before largely levelling off above 12 THz. The slower rate of increase, particularly in the *xy* plane, could be explained by the avoided crossings in the phonon dispersion discussed in Section 2.3.

To decrease the *κ*_l_, one would need to reduce the group velocities and/or lifetimes of the low-frequency modes. To achieve this heavier and larger substitutional point defects should be introduced into the structure to create greater variation in the ion sizes in the crystal lattice, which would act as scattering centres and promote “electron-crystal, phonon-glass” behaviour. As shown in Fig. 5a and 12a, modes involving the Y atoms predominantly occur below 4 THz, so substituting Y with a heavier atom of similar electronegativity and bonding, for example La, would likely decrease the *κ*_l_. Alternatively, substituting S in BiCuOS for Se in BiCuOSe has been shown to substantially improve the *ZT*.^57^ Following that argument, we speculate that moving from S to Se could potentially lower the lattice thermal conductivity and thus offer increased thermoelectric performance in Y_2_Ti_2_O_5_Se_2_ compared to Y_2_Ti_2_O_5_S_2_. Y_2_Ti_2_O_5_Se_2_ has not been experimentally realised or computationally predicted thus far, and so perhaps only a partial replacement of S by Se is achievable.

The cumulative *κ*_l_ can also be examined as a function of the phonon mean free path (MFP), *Λ*_λ_ = *υ*_λ_*τ*_λ_. This is shown for the in-plane and out-of-plane directions at 300 K and 1000 K in Fig. 12b and c, respectively. At high temperature, the phonon MFPs in Y_2_Ti_2_O_5_S_2_ are short, with only 6% of the phonon modes possessing MFPs longer than 20 nm in either direction. A slightly larger proportion of modes have MFPs longer than 10 nm – 17% in the *xy* plane and 14% along the *z* direction. At 300 K, longer phonon lifetimes are observed, such that 25% of the *κ*_l_ arises from modes with MFPs above 20 nm and 40% from modes with MFPs longer than 10 nm. This suggests that nanostructuring to limit the phonon mean-free paths could in principle reduce the *κ*_l_, in particular at lower temperature, which we explore in the following subsection.

### 3.2 Nanostructuring

Nanostructuring is commonly employed as a strategy to decrease the lattice thermal conductivity, as phonons with long MFPs can be scattered at surfaces and interfaces (*e.g.* grain boundaries) to limit the transport through these modes. To simulate the effects of nanostructuring, we introduced a boundary scattering term of *υ*_λ_/*Λ*_λ_. Based on this model, we predict that nanostructuring to dimensions smaller than 20 nm would decrease the in-plane and out-of-plane lattice thermal conductivities, respectively, from 2.27 W m^−1^ K^−1^ and 0.76 W m^−1^ K^−1^ to 1.87 W m^−1^ K^−1^ and 0.66 W m^−1^ K^−1^ at 1000 K. Nanostructuring to 10 nm is predicted to have a larger effect, reducing the thermal conductivity along the two directions to 1.64 W m^−1^ K^−1^ and 0.53 W m^−1^ K^−1^. A larger reduction is predicted at lower temperatures, particularly below 300 K, where nanostructuring decreases the thermal conductivity by over a third.

Nanostructuring also affects the electron mean-free paths, so the same boundary-scattering formalism was applied to investigate the impact on the electrical transport properties. The inclusion of the additional scattering term is expected to decrease the conductivity, power factor and electronic thermal conductivity. On the other hand, the total thermal conductivity, *i.e.* the sum of lattice and electronic thermal conducitivities, is expected to decrease, which may compensate for the decreased power factor to increase the overall *ZT*.

For grain sizes of 10 nm and 20 nm the transport properties follow the expected trends across all temperatures and charge carrier concentrations. At charge carrier concentrations below 4 × 10^19^ cm^−3^, the predicted decrease in the power factor is less than 30%. The greatest percentage reductions are seen for high carrier concentrations and at low temperatures where the power factors are generally low. In absolute terms, the maximum reduction of over 3200 μW m^−1^ K^−2^ is observed for carrier concentrations between 2.37 × 10^20^ cm^−3^ to 4.22 × 10^20^ cm^−3^ and temperatures between 350 K to 625 K for a grain size of 10 nm. A smaller reduction is predicted for a grain size of 20 nm, for which the power factor decreases by at most by 2400 μW m^−1^ K^−2^ between *n* = 2.37 × 10^20^ cm^−3^ to 4.22 × 10^20^ cm^−3^ and *T* = 350 K to 575 K.

The question then becomes whether the reduction in the lattice thermal conductivity can offset the decreased power factor to increase the *ZT*. While a 10% increase in the *ZT* is observed along the *z* direction, the very low conductivity and power factor mean the *ZT* remains around 0.01. In the *xy* plane, the maximum *ZT* is in fact decreased on limiting the MFPs, as can be seen in Table 4: the more favourable (*i.e.* lower) thermal conductivity obtained by nanostructuring is mitigated by the reduced power factor.

**Table 4:** Maximum predicted in-plane and out-of-plane *ZT* at 1000 K and the corresponding lattice (*κ*_l_) and electronic (*κ*_e_) thermal conductivity, charge carrier concentration (*n*) and power factor (PF). Also shown are the values obtained with simulated grain sizes of 10 nm and 20 nm

Direction	MFP (nm)	*ZT*	*κ*_l_ (W m^−1^ K^−1^)	*κ*_e_ (W m^−1^ K^−1^)	*n* (cm^−3^)	PF (μW m^−1^ K^−2^)
*xy*	—	1.18	2.27	1.60	2.37 × 10^20^	4556
*z*	—	0.013	0.76	0.006	1 × 10^21^	10
*xy*	10	1.09	1.64	0.79	1.77 × 10^20^	2647
*xy*	20	1.13	1.87	0.91	1.77 × 10^20^	3108

**Fig. 13:**
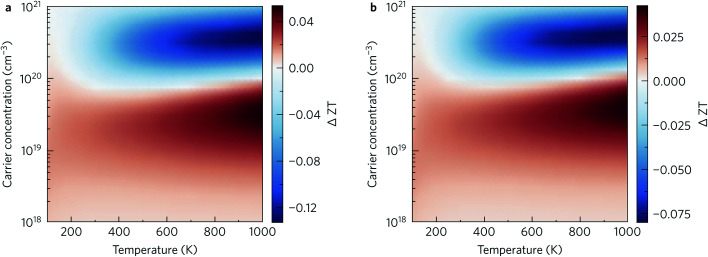
Difference in the thermoelectric figure of merit *ZT* obtained by nanostructuring to limit the mean-free paths to (a) 10 nm and (b) 20 nm. Positive (red) and negative (blue) areas indicate that the *ZT* is enhanced and reduced, respectively, by the nanostructuring.

That said, at lower charge-carrier concentrations our calculations predict that nanostructuring would improve the *ZT*. Fig. 13 shows the absolute difference in the *ZT* obtained by nanostructuring to both 10 nm (Figure 13a) and 20 nm (Fig. 13b), which shows similar trends. Below carrier concentrations of 10^20^ cm^−3^, a slight increase in *ZT* is seen, with larger increases at higher temperatures. On the other hand, the scattering introduced by grain boundaries at higher charge-carrier concentrations leads to a decrease in the power factor that is not compensated by the decrease in the thermal conductivity. We noted earlier that maximising the *ZT* requires doping levels that are close to the limit of what is routinely achievable in experiments – we therefore conclude that the utility of nanostructuring for optimising the *ZT* of this material depends on the doping levels achievable in experiments.

## 4 Conclusions

In conclusion, we have demonstrated using state-of-the-art DFT calculations that Y_2_Ti_2_O_5_S_2_ is a promising high- and intermediate-temperature n-type thermoelectric with a predicted maximum in-plane *ZT* of 1.18 at 1000 K, which is obtained for a carrier concentration of 2.37 × 10^20^ cm^−3^.

The electronic structure, lattice dynamics and transport properties show significant anisotropy, which arises from the unique quasi-layered crystal structure. The anisotropy presents most clearly in the electronic band structure, where the flat bands along the real-space *z* direction inhibit the electrical conductivity and lead to a *ZT* that is two orders of magnitude lower compared to the in-plane figure of merit. This is despite the lower lattice thermal conductivity, which is predicted to be ∼30% smaller along the out-of-plane direction than in the *xy* plane.

The large *ZT* is also driven by the intrinsically-low lattice thermal conductivities of 3.77 W m^−1^ K^−1^ at 600 K and 2.27 W m^−1^ K^−1^ at 1000 K. Due to the short phonon mean free paths, we predict that nanostructururing is unlikely to be a viable strategy for enhancing the *ZT*, as this only decreases the lattice thermal conductivity by 6% at 1000 K but decreases the power factor by an average of 18% depending on the charge-carrier concentration. On the other hand, we would anticipate that alloying with La or Se, or doping with extrinsic dopants such as halides, could decrease the lattice thermal conductivity without negatively impacting the power factor.

To investigate this further, work is currently underway to establish the defect chemistry and to confirm whether Y_2_Ti_2_O_5_S_2_ can be doped to the required charge-carrier concentrations to achieve the high *ZT*s predicted in this study.

## Conflicts of interest

There are no conflicts to declare.

## Supplementary Material

TA-010-D2TA04160J-s001
