# Supplementary material for: Y2Ti2O5S2 – a promising n-type oxysulphide for thermoelectric applications
Source: J Mater Chem A Mater. 2022 Jul 4;10(32):16813–24. doi: 10.1039/d2ta04160j (PMC9382646; doi:10.1039/d2ta04160j)
Supplement: TA-010-D2TA04160J-s001 [file TA-010-D2TA04160J-s001.pdf]

Electronic Supplementary Material (ESI) for Journal of Materials Chemistry A.
This journal is © The Royal Society of Chemistry 2022

Supporting information for: $Y_2Ti_2O_5S_2$ – a promising n-type oxysulphide for thermoelectric applications

Katarina Brlec, Kieran B. Spooner, Jonathan M. Skelton, David O. Scanlon

June 9, 2022

1 Energy cutoff and k-point mesh convergence testing

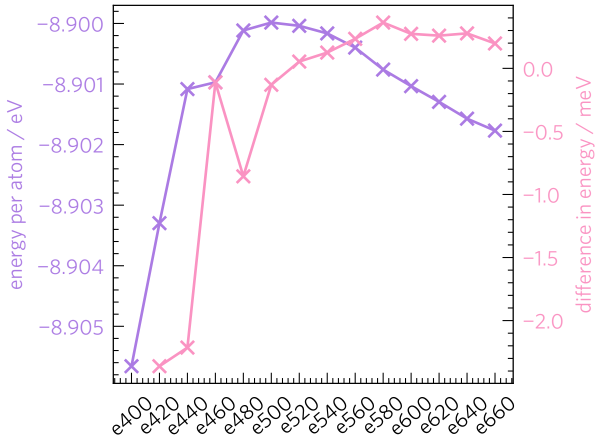

Figure S1: Convergence of the total energy of the primitive cell with respect to the plane-wave cutoff. The energy cutoff selected for the calculations was 480 eV.

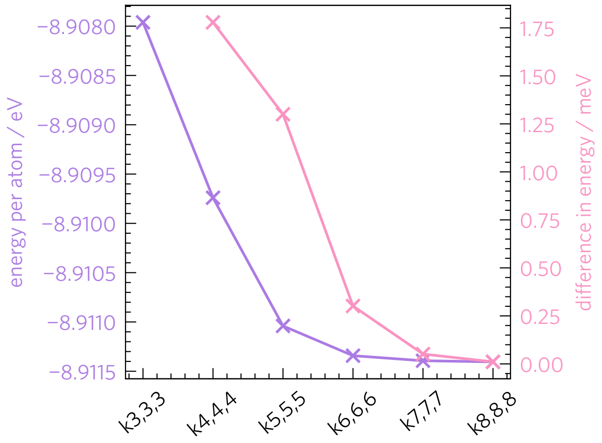

Figure S2: Convergence of the total energy of the primitive cell with respect to k-point sampling density. A k-point mesh with $5 \times 5 \times 5$ subdivisions was selected for the calculations.

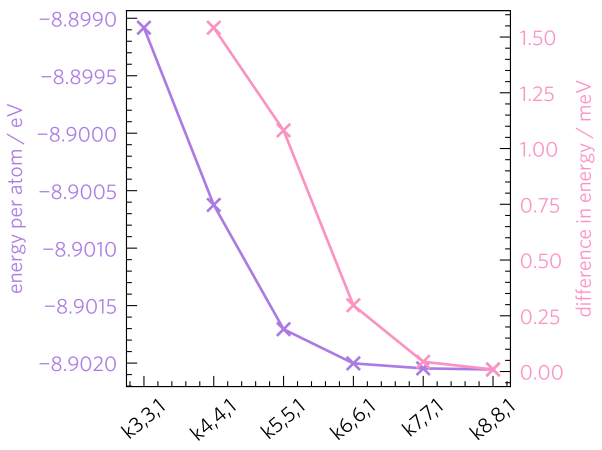

Figure S3: Convergence of the total energy of the primitive cell with respect to k-point sampling density. A k-point mesh with $5 \times 5 \times 1$ subdivisions was selected for the calculations.

2 Brillouin zone

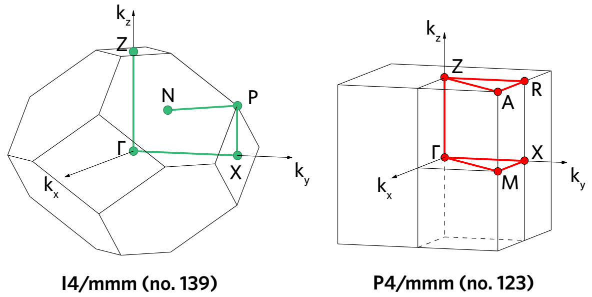

Figure S4: Reciprocal spaces of the $I4/mmm$ and $P4/mmm$ space groups as per Bradley-Cracknell formalism.[1]

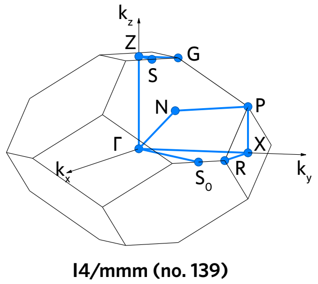

Figure S5: Reciprocal space of the $I4/mmm$ space group as per seekpath formalism.[2]

3 Extended electronic band structure

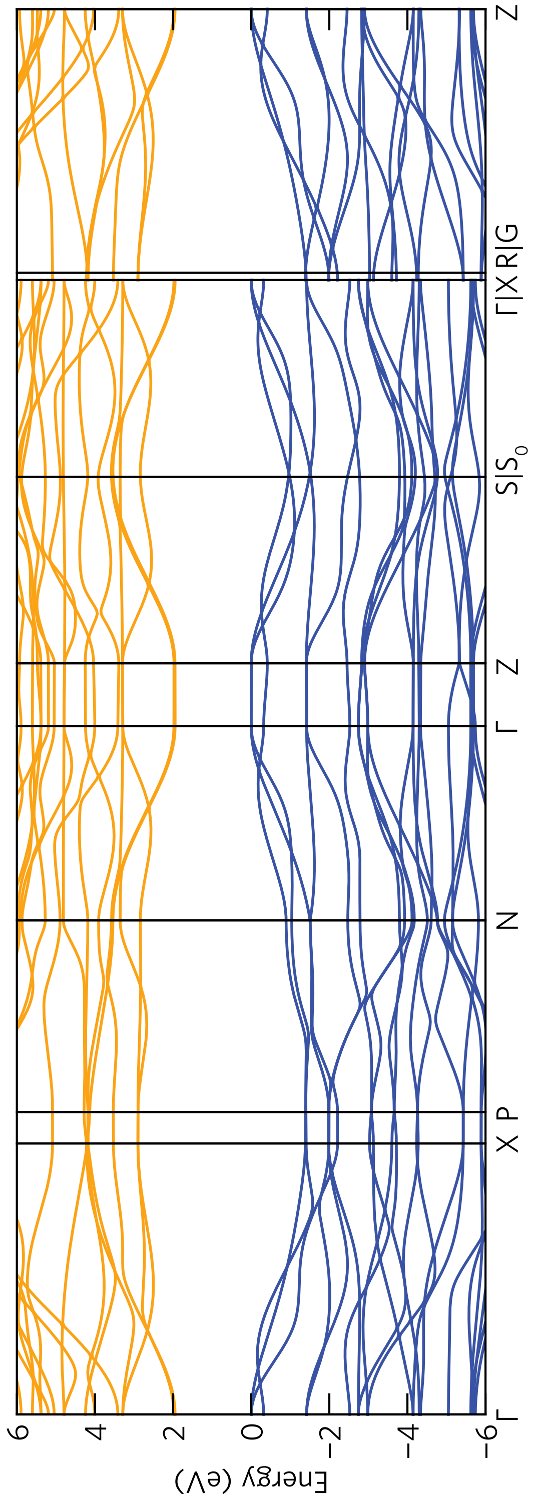

Figure S6: Electronic band structure calculated using HSE06 and the SeeK-path band path, which contains additional wavevectors compared to the Bradley-Cracknell path.[2]

4 Band alignment calculations

The band alignment was calculated according to the core-vacuum alignment scheme derived by Wei and Zunger.[3] The ionisation potential (IP) and electron affinity (EA) were calculated as:

$$IP = (E_{vac} - E_{core,slab}) - (E_{VBM} - E_{core,bulk}) \tag{1}$$

$$EA = IP - E_g \tag{2}$$

where E_{vac} and $E_{core,slab}$ are the energies of the vacuum and the O 1s core level in the bulk-like surface slab, respectively, E_{VBM} is the valence band maximum of the bulk and $E_{core,bulk}$ is the bulk O 1s core energy.

To calculate the energies of the surface slab, the surfaxe[4] package was used to cleave the (001) slab from the HSE06-relaxed conventional unit cell. As we are interested in the band alignment of the bulk, the slab was not relaxed and only a static calculation was performed.

5 Phonon supercell convergence testing

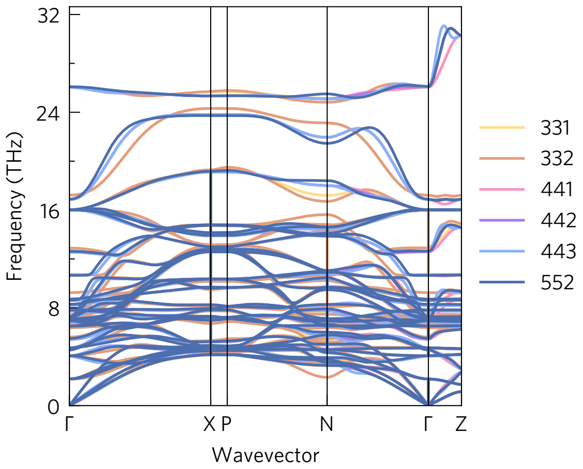

Figure S7: Phonon dispersions calculated from second-order force constants obtained in $3 \times 3 \times 1$, $3 \times 3 \times 2$, $4 \times 4 \times 1$, $4 \times 4 \times 2$, $4 \times 4 \times 3$ and $5 \times 5 \times 2$ supercells, plotted with ThermoPlotter.[5] The high-symmetry path is based on the Bradley-Cracknell formalism.[1]

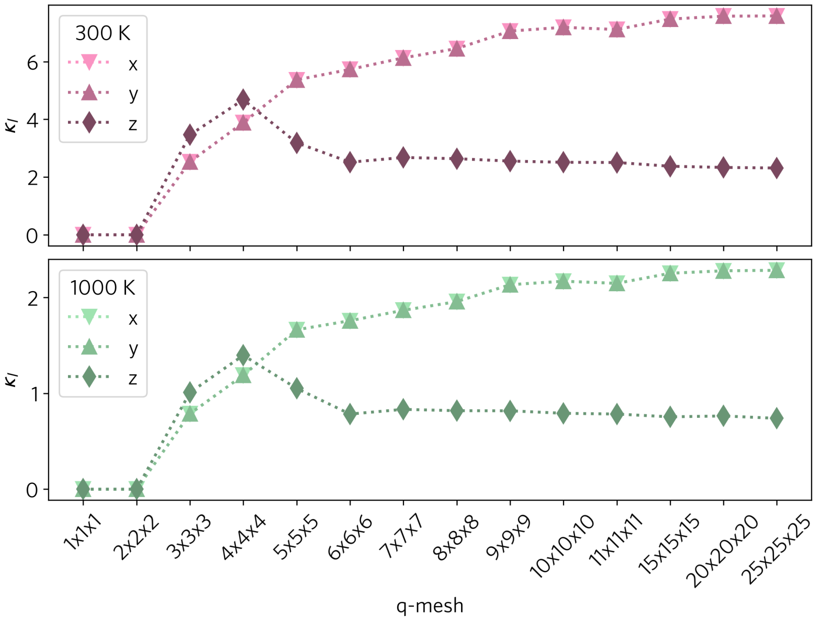

Figure S8: Convergence of the lattice thermal conductivity κ_l convergence at 300 K and 1000 K with respect to the q-point mesh sampling density.

6 Projection of the symmetry inequivalent O eigen-displacements onto the phonon dispersion

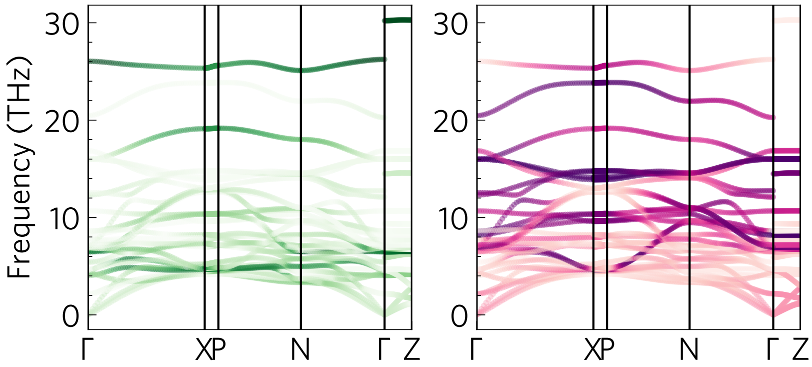

Figure S9: Phonon dispersion computed using a 5 × 5 × 2 supercell expansion showing the projection of the mode eigen-displacements onto the axial (a, green) and equatorial O atoms (b, purple). The darker the colour, the greater the contribution from those eigen-displacements. The high-symmetry paths were created using Bradley-Cracknell formalism.[1]

7 AMSET settings

Polar optical phonon frequency: 8.51 THz

Elastic constant matrix (GPa):

$$\begin{bmatrix} 283.017 & 88.337 & 108.044 & 0 & 0 & 0 \\ 88.337 & 283.017 & 108.044 & 0 & 0 & 0 \\ 108.044 & 108.044 & 222.213 & 0 & 0 & 0 \\ 0 & 0 & 0 & 46.486 & 0 & 0 \\ 0 & 0 & 0 & 0 & 46.486 & 0 \\ 0 & 0 & 0 & 0 & 0 & 109.067 \end{bmatrix} \tag{3}$$

Static dielectric constant (ϵ_0):

$$\begin{bmatrix} 36.665 & 0 & 0 \\ 0 & 36.665 & 0 \\ 0 & 0 & 23.630 \end{bmatrix} \tag{4}$$

High-frequency dielectric constant (ϵ_0):

$$\begin{bmatrix} 5.361 & 0 & 0 \\ 0 & 5.361 & 0 \\ 0 & 0 & 5.489 \end{bmatrix} \tag{5}$$

8 Scattering rates

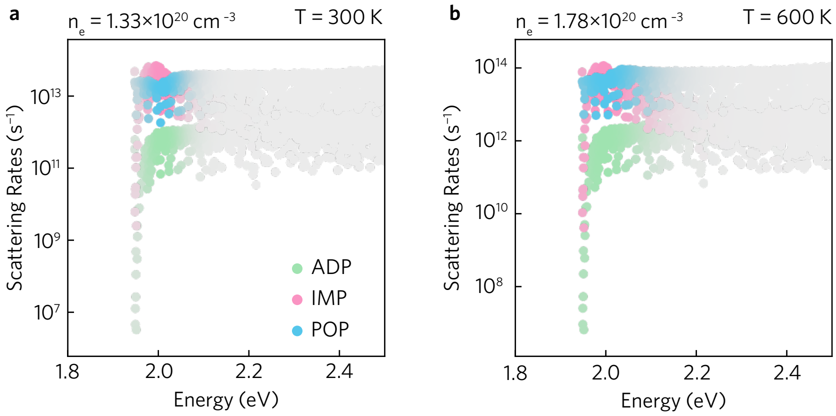

Figure S10: Scattering rates as a function of energy for carrier concentrations and temperatures of (a) $n = 1.33 \times 10^{20}\,\mathrm{cm}^{-3}$ and $T = 300\,\mathrm{K}$ and (b) $n = 1.78 \times 10^{20}\,\mathrm{cm}^{-3}$ and $T = 600\,\mathrm{K}$. The marker colours are weighted by Fermi-Dirac distribution such that those with darker colours make a larger contribution to the transport properties. Acoustic deformation potential (ADP) scattering is shown in green, ionisation impurity scattering (IMP) scattering in pink and polar optical phonon (POP) scattering in blue.

References

[1] C. J. Bradley and A. P. Cracknell, *The Mathematical Theory of Symmetry in Solids: Representation Theory for Point Groups and Space Groups*, Clarendon Press, Oxford, 1972.

[2] Y. Hinuma, G. Pizzi, Y. Kumagai, F. Oba and I. Tanaka, *Computational Materials Science*, 2017, **128**, 140–184.

[3] S.-H. Wei and A. Zunger, *Appl. Phys. Lett.*, 1998, **72**, 2011–2013.

[4] K. Brlec, D. W. Davies and D. O. Scanlon, *J. Open Source Softw.*, 2021, **6**, 3171.

[5] K. B. Spooner, M. Einhorn, D. W. Davies and D. O. Scanlon, *ThermoPlotter*, Online, 2021, https://github.com/SMTG-UCL/ThermoPlotter.
